# Pre-Existing Adenovirus Immunity Modifies a Complex Mixed Th1 and Th2 Cytokine Response to an Ad5/HIV-1 Vaccine Candidate in Humans

**DOI:** 10.1371/journal.pone.0018526

**Published:** 2011-04-13

**Authors:** Samuel O. Pine, James G. Kublin, Scott M. Hammer, Joleen Borgerding, Yunda Huang, Danilo R. Casimiro, M. Juliana McElrath

**Affiliations:** 1 Program in Pathobiology, Department of Global Health, University of Washington, Seattle, Washington, United States of America; 2 Department of Laboratory Medicine, University of Washington, Seattle, Washington, United States of America; 3 Vaccine and Infectious Disease Division, Fred Hutchinson Cancer Research Center, Seattle, Washington, United States of America; 4 Division of Infectious Diseases, Columbia University College of Physicians and Surgeons, New York, New York, United States of America; 5 Vaccine Basic Research, Merck Research Laboratories, West Point, Pennsylvania, United States of America; Massachusetts General Hospital, United States of America

## Abstract

The results of the recent Step Study highlight a need to clarify the effects of pre-existing natural immunity to a vaccine vector on vaccine-induced T-cell responses. To investigate this interaction, we examined the relationship between pre-existing Ad5 immunity and T-cell cytokine response profiles in healthy, HIV-uninfected recipients of MRKAd5 HIV-1 gag vaccine (HVTN 050, ClinicalTrials.gov #NCT00849732). Participants were grouped by baseline Ad5 neutralizing antibody titer as either Ad5-seronegative (titer ≤18; n = 36) or Ad5-seropositive (titer >200; n = 34). Samples from vaccine recipients were analyzed for immune responses to either HIV-1 Gag peptide pools or Ad5 empty vector using an *ex vivo* assay that measures thirty cytokines in the absence of long-term culture. The overall profiles of cytokine responses to Gag and Ad5 had similar combinations of induced Th1- and Th2-type cytokines, including IFN-γ, IL-2, TNF-α, IP-10, IL-13, and IL-10, although the Ad5-specific responses were uniformly higher than the Gag-specific responses (p<0.0001 for 9 out of 11 significantly expressed analytes). At the peak response time point, PBMC from Ad5-seronegative vaccinees secreted significantly more IP-10 in response to Gag (p = 0.008), and significantly more IP-10 (p = 0.0009), IL-2 (p = 0.006) and IL-10 (p = 0.05) in response to Ad5 empty vector than PBMC from Ad5-seropositive vaccinees. Additionally, similar responses to the Ad5 vector prior to vaccination were observed in almost all subjects, regardless of Ad5 neutralizing antibody status, and the levels of secreted IFN-γ, IL-10, IL-1Ra and GM-CSF were blunted following vaccination. The cytokine response profile of Gag-specific T cells mirrored the Ad5-specific response present in all subjects before vaccination, and included a number of Th1- and Th2-associated cytokines not routinely assessed in current vaccine trials, such as IP-10, IL-10, IL-13, and GM-CSF. Together, these results suggest that vector-specific humoral responses may reduce vaccine-induced T-cell responses by previously undetected mechanisms.

## Introduction

Most T-cell-targeted HIV vaccine candidates are based on vectors derived from naturally occurring human viruses, and pre-existing immunity to these viruses has the potential to negatively impact the desired vaccine response. For instance, the recent Step Study HIV T-cell vaccine test-of-concept/efficacy trial unexpectedly showed that vaccine recipients who had pre-existing neutralizing antibodies against the vaccine's non-replicating adenovirus serotype 5 (Ad5) vector trended toward having an increased risk of HIV-1 infection [1]. While it has been shown that Ad5-specific antibodies blunt the desired immune response to an Ad5-based vaccine in a dose-dependent manner [2], [3], [4], it remains unclear how this may translate into an increased risk of HIV-1 infection.

Ad5-specific antibodies limit the dose and duration of target cell exposure to Ad5-vaccine viral particles, which lower immune responses by reducing the frequency of transduced cells [3]. Such restriction is particularly vexing because Ad5 seropositivity rates tend to be higher in populations with higher risks of HIV infection [5], [6]. Blunting of a vaccine response due to neutralizing antibodies can be partially overcome by increasing the dose or number of inoculations [3], [6], but even with such dose optimization, rates of HIV-specific IFN-γ T-cell responses induced by Ad5/HIV-1 vaccination remain higher in vaccine recipients with lower Ad5 neutralizing antibody titers [1], [2]. However, in the Step Study, the IFN-γ responses were similar in HIV-1 infected and uninfected vaccine recipients who were positive for Ad5 neutralizing antibodies, suggesting that lower IFN-γ response rates cannot be solely responsible for the increased trend in infection rates for Ad5-seropositive vaccine recipients. In addition, *in vitro* studies indicate that vector-specific antibodies can enhance *trans* infection of T cells by HIV-1 via the formation of immune complexes [7] and Ad5-specific CD4^+^ T-cell proliferative responses correlate with increased expression of mucosal homing markers [8], but evidence for such an interaction *in vivo* remains to be demonstrated.

Antiviral T-cell responses are not comprised of a single function and often include the simultaneous production of multiple cytokines, which can indicate varying capacities for proliferative, immunostimulatory, and cytotoxic effector functions [9], [10], [11], [12]. Comparisons between infected cases and non-infected matched controls among Step Study vaccine recipients showed no significant differences in terms of HIV-specific T-cell IFN-γ, IL-2, and TNF-α responses by T-cell subset, breadth, magnitude, or polyfunctional profile [2]. In addition, no differences were observed between these groups in the frequency of total peripheral CD4^+^CCR5^+^ T cells or activated Ki67^+^Bcl-2^−^ HIV-1 target cells. However, lower Ad5-specific CD4^+^ T-cell responses were detected in HIV-1-infected cases when compared to non-infected matched controls [2]. Thus, even though polyfunctional T cells have been correlated with favorable HIV-1 disease outcomes [13], reliable correlates of immune protection remain elusive.

Based on the lower Ad5-specific CD4^+^ T-cell response rates observed in Step Study cases and the lack of observed differences in HIV-specific T-cell responses, we hypothesized that pre-existing Ad5-neutralizing antibody titers influence vaccine-induced cytokine production of HIV-1- and Ad5-specific responses, but that current multi-parameter flow cytometric techniques may not be sufficient to measure these differences. Since characterizing T-cell responses by flow cytometry is presently limited to concurrent measurement of four intracellular cytokines [14], we devised a novel T-cell response assay to simultaneously measure thirty secreted factors from *ex vivo*-stimulated peripheral blood mononuclear cells (PBMC) without clonal expansion. By using multiplexed bead arrays to explore T-cell cytokine responses, we could concurrently measure factors linked to a variety of immune functions, such as Th1, Th2, pro-inflammatory, and chemotactic responses. Using this method, we examined the cellular immune responses of Ad5-seronegative and Ad5-seropositive volunteers pre- and post-vaccination with a MRKAd5 HIV-1 gag vaccine similar to the one evaluated in the Step Study. We determined that Ad5-specific immunity contributes significantly to the vaccine-induced response, and that certain factors of the T-cell response profile are associated with pre-existing Ad5 neutralizing antibody titers.

## Materials and Methods

### Ethics Statement

All volunteers provided informed written consent prior to enrollment in the HVTN 050 clinical trial (ClinicalTrials.gov #NCT00849732). The trial was approved by the institutional/human subjects review boards of the sponsor institutions and the trial clinical sites: Hospital Escola São Francisco de Assis (Rio de Janeiro, Brazil), Centro de Referencia e Treinamento (São Paulo, Brazil), Federal University of Sao Paulo (São Paulo, Brazil), Cornell–GHESKIO (Port-au-Prince, Haiti), IMPACTA (Lima, Perú), Maternal Infant Studies Center (San Juan, Puerto Rico), Research Institute for Health Sciences (Chiang Mai, Thailand), Mahidol University (Bangkok, Thailand), AFRIMS (Bangkok, Thailand). University of Alabama at Birmingham (Birmingham, AL, USA), San Francisco Department of Public Health (San Francisco, CA, USA), Johns Hopkins University (Baltimore, MD, USA), University of Maryland at Baltimore (Baltimrore, MD, USA), Brigham & Women's Hospital (Boston, MA, USA), Fenway Community Health (Boston, MA, USA), Harvard University/Brown University (Boston, MA, USA), Saint Louis University School of Medicine (St. Louis, MO, USA), New York Blood Center, Columbia University (New York City, NY, USA), University of Rochester Medical Center (Rochester, NY, USA), Vanderbilt University (Nashville, TN, USA) and Fred Hutchinson Cancer Research Center/University of Washington (Seattle, WA, USA).

### Study population and vaccine regimen

HIV-uninfected individuals between the ages of 19 and 50 years old were enrolled in a multi-center, double-blind, placebo-controlled HIV-1 vaccine trial of the MRKAd5 HIV-1 gag monovalent vaccine candidate, the results of which have been previously reported [15]. This vaccine contained a non-replicating Ad5 vector based on an adenovirus Group C strain with the E1 region replaced with a clade B codon-optimized HIV-1_CAM-1_ *gag* sequence [16], [17], [18]. Participants randomized to the vaccine arm received either 10^9^ or 10^10^ viral particles of the MRKAd5 HIV-1 gag vaccine intramuscularly at 0, 4, and 26 weeks. After study unblinding, vaccine recipients (n = 70) who mounted vaccine-induced IFN-γ-secreting Gag-specific T-cell responses were chosen, as determined by IFN-γ ELISpot at 30 weeks after the first vaccination (median, 352 spot forming cells (SFC)/10^6^ PBMC; interquartile range (IQR), 56–2481). The vaccinated subjects were stratified into two groups based upon their pre-immunization (baseline) Ad5 neutralizing antibody titer: participants with baseline Ad5 neutralizing antibody titers ≤18 (n = 36) defined as the Ad5-seronegative group, while those with baseline Ad5 neutralizing antibody titers >200 (n = 34) were defined as the Ad5-seropositive group. The two Ad5-serostatus groups were matched in regard to the number of subjects included who received either 10^9^ or 10^10^ viral particles dose of vaccine (30% 10^9^ VPU vs. 70% 10^10^ VPU). The population demographics for the test groups used in this study are shown in Table 1.

**Table 1:** Demographic characteristics of study populations.

	*Ad5-seronegative (n = 36)*	*Ad5-seropositve (n = 34)*	*p-value^1^*
**Median Age (range)**	31 (19–47)	27 (19–49)	0.33
**Gender n, (%)**			0.16
** Male**	24 (66.7)	17 (50.0)	
** Female**	12 (33.3)	17 (50.0)	
**Race/Ethnicity n, (%)**			0.001
** Caucasian**	19 (52.8)	10 (29.4)	
** African/Black**	6 (16.7)	3 (8.8)	
** Asian**	1 (2.8)	14 (41.2)	
** Hispanic**	7 (19.4)	4 (11.8)	
** Other**	3 (8.3)	3 (8.8)	
**Region n, (%)**			0.001
** U.S.**	21 (58.3)	8 (23.5)	
** Thailand**	1 (2.8)	13 (38.2)	
** S. America**	10 (27.8)	10 (29.4)	
** Caribbean**	4 (11.1)	3 (8.8)	
**Median IFN-**γ **secreting cells** 2			0.42
** SFC/10^6^ PBMC (range)**	362 (56–2481)	292 (75–1179)	
**Median nAb Ad5 Titer (range)**			
** Pre-vaccination**	≤18 (−)	515 (217−≥4608)	<0.0001
** Peak response**	447 (26−≥4608)	2821 (488−≥4608)	<0.0001

1 Wilcoxon rank-sum test used for age, ELISpot and Ad5 titer comparisons; Chi-square test used for comparing race/ethnicity and region.

2 ELISpot results exclude two Ad5-naïve and seven Ad5-immune vaccinees due to the use of alternate peptide pools for stimulations.

In addition, assay validation procedures were conducted in PBMC from chronically HIV-1-infected (n = 5) and HIV-1-uninfected (n = 24) volunteers. The HIV-seropositive control samples were selected based on previously detected Gag-specific IFN-γ ELISpot responses, whereas the HIV-seronegative control samples were isolated from HIV-1 low-risk, healthy volunteers. These studies were approved by the institutional human subjects review board at each clinical site prior to study initiation, and all vaccine recipients and control group subjects provided a written informed consent prior to participation.

### Sample collection and Ad5 serology

Anticoagulated blood from vaccine recipients was collected by venipuncture into standard EDTA collection tubes, and PBMC were isolated on a density gradient and cryopreserved on the day of collection as previously described [19]. PBMC were collected for immunogenicity testing prior to vaccination (baseline) and at peak time points (28 and 30 weeks after the first vaccination). Serum samples were collected for Ad5 serology testing at a pre-vaccination screening visit and at 4-, 8-, 12-, 30-, 42-, and 78-week time points after administration of the first vaccine dose. Ad5 neutralizing antibody titers in serum were determined ≤45 days prior to the first immunization as previously described [6].

### IFN-γ ELISpot assays

Unfractionated PBMC were assayed by a validated IFN-γ ELISpot assay as previously described [20], using overlapping 15-mer peptide pools based on the homologous HIV-1 *gag* vaccine sequence to determine antigen-specific responses. Results were reported as the number of spot forming cells (SFC) per 10^6^ input cells. Positive responses were defined as ≥55 SFC/10^6^ cells and a ≥4-fold response over negative control wells.

### Ex vivo multiplex cytokine assay

Cryopreserved PBMC from vaccinees were thawed and incubated overnight at 37°C, 5% CO_2_, and 95% humidity in R10 media (RPMI 1640 [Invitrogen, San Diego, CA] supplemented with 10% fetal bovine serum [Gemini Bio-Products, West Sacramento, CA], 2 mM L-glutamine, 100 U/ml penicillin, and 100 µg/ml streptomycin [Invitrogen]). Cells were plated the following day in triplicate at a concentration of 2×10^5^ cells/well in round-bottom plates. PBMC were stimulated with either 1µg/ml of pooled 15-mer peptides representing the potential T-cell epitopes (PTE) present in all global Gag sequences [21], a non-replicating E1/E3 deletion mutant of Ad5 at an MOI of 25,000, or an equivalent volume of DMSO, and were incubated for 48 hours, after which supernatants were collected, aliquoted, and frozen at −80°C. This incubation time was pre-determined to be optimal for detecting maximal expression of the analytes being measured while minimizing loss due to *in vitro* consumption (data not shown). Cytokines and chemokines were quantified using a Human Lincoplex 30-plex bead array kit (Linco, St. Charles, MO) modified from the manufacturer's instructions to provide higher statistical power for curve fitting algorithms by applying a nine-point standard curve rather than the recommended six-point standard curve (see Table 2 for a complete list of the thirty cytokines and chemokines analyzed). Data were collected using a Luminex 200 system (Perkin Elmer, Wellesley, MA) with MiraiBio Masterplex acquisition and analysis software (Hitachi Software Engineering, South San Francisco, CA). A five-parameter logistic curve fit was used with 1/Y weighting to establish analyte-specific concentrations. The geometric mean of the triplicate control wells was used as a measure of background for calculating either the fold-change or background-subtracted concentration for each participant and stimulation condition.

**Table 2:** Response rate and magnitude of response to Gag peptides or Ad5 empty vector at peak timepoint after three vaccine doses.

		*Response Rate n = 56 (%)*		*Median Concentration for Responders in pg/ml (IQR)*			*p-value^2^*	
*Functional Category*	*Analyte^1^*	*Gag*	*Ad5*	*Control*	*Gag*	*Ad5*	*Control vs. Gag*	*Control vs. Ad5*
Th1-type	**IFN-**γ	**48 (86)**	**55 (98)**	**5 (1**–**15)**	**134 (69**–**276)**	**280 (163**–**493)**	**<0.001**	**<0.001**
	**IL-2**	**49 (88)**	**56 (100)**	**3 (1**–**5)**	**33 (21**–**60)**	**81 (51**–**113)**	**<0.001**	**<0.001**
	**TNF-α**	**36 (64)**	**42 (75)**	**18 (12**–**41)**	**44 (25**–**60)**	**43 (30**–**75)**	**<0.001**	**<0.001**
	IL-7	26 (46)	30 (54)	17 (14–26)	22 (19–26)	22 (18–26)	<0.001	<0.001
	IL-15	8 (14)	2 (4)	1 (1–2)	6 (4–9)	2 (1–3)	0.008	NA
	**sCD40L**	**26 (46)**	**44 (79)**	**1 (1**–**4)**	**13 (9**–**19)**	**33 (23**–**50)**	**<0.001**	**<0.001**
Th2-type	IL-4	8 (14)	10 (18)	1 (1–17)	15 (8–17)	20 (14–30)	0.25	0.002
	IL-5	NA	NA	NA	NA	NA	NA	NA
	**IL-13**	**40 (71)**	**50 (89)**	**3 (1**–**8)**	**37 (22**–**67)**	**60 (43**–**88)**	**<0.001**	**<0.001**
Immuno-modulatory	**IL-10**	**29 (52)**	**53 (95)**	**73 (48**–**173)**	**128 (100**–**155)**	**605 (344**–**953)**	**0.5**	**<0.001**
	**IL-17**	**19 (34)**	**32 (57)**	**2 (1**–**3)**	**4 (3**–**5)**	**5 (3**–**6)**	**0.012**	**<0.001**
	**IL-1Ra**	**21 (38)**	**41 (73)**	**296 (179**–**426)**	**633 (318**–**1101)**	**681 (477**–**1391)**	**<0.001**	**<0.001**
	IL-12p40	4 (7)	0 (0)	15 (1–30)	16 (9–22)	NA	NA	NA
	IL-12p70	6 (11)	4 (7)	1 (1–1)	1 (1–2)	1 (0–2)	0.13	NA
Pro-inflammatory	IL-1α	23 (41)	20 (36)	44 (28–88)	87 (65–131)	82 (57–98)	0.13	0.021
	IL-1β	24 (43)	27 (48)	13 (5–56)	10 (5–22)	6 (3–12)	<0.001	<0.001
	IL-6	15 (27)	15 (27)	172 (63–3684)	562 (162–3218)	584 (133–1947)	0.98	0.19
Chemokines	IL-8	33 (59)	29 (52)	8036 (6228–9705)	9001 (7707–10000)	9675 (8671–10000)	<0.001	<0.001
	MIP-1α	14 (25)	29 (52)	299 (10–1389)	66 (28–718)	133 (91–378)	0.14	0.62
	**MIP-1**β	**27 (48)**	**33 (59)**	**179 (103**–**545)**	**328 (190**–**485)**	**524 (357**–**704)**	**0.036**	**0.019**
	RANTES	1 (2)	9 (16)	470 (470–470)	51 (51–51)	49 (34–165)	NA	0.36
	**IP-10**	**56 (100)**	**54 (96)**	**18 (6**–**43)**	**810 (239**–**1764)**	**955 (289**–**2022)**	**<0.001**	**<0.001**
	MCP-1	11 (20)	12 (21)	1707 (687–3864)	5416 (3707–10000)	4490 (3575–7574)	0.019	<0.001
	Fractalkine	NA	NA	NA	NA	NA	NA	NA
	Eotaxin	1 (2)	0 (0)	11 (11–11)	8 (8–8)	NA	1.0	NA
Growth factors	G-CSF	22 (39)	16 (29)	59 (25–247)	128 (67–278)	145 (46–430)	0.003	0.56
	**GM-CSF**	**39 (70)**	**47 (84)**	**28 (21**–**69)**	**78 (50**–**136)**	**76 (55**–**114)**	**<0.001**	**<0.001**
	EGF	6 (11)	4 (7)	5 (1–6)	5 (4–6)	1 (1–3)	0.56	NA
	TGF-α	21 (38)	20 (36)	3 (1–5)	4 (3–7)	6 (3–8)	0.002	0.23
	VEGF	18 (32)	26 (46)	10 (3–34)	13 (9–23)	19 (16–25)	0.77	0.61

1 Bold denotes analytes with ≥50% response rate to either Gag or Ad5 with concentrations significantly 2-fold higher or lower than controls.

2 Wilcoxon sign-rank two-tailed test using responders when n ≥5 for each group.

Abbreviations: NA, not applicable.

To validate the specificity of this assay, we measured the 30-plex cytokine response of HIV-1 Gag-stimulated PBMC from 24 healthy, HIV-seronegative volunteers (n = 19 from vaccine trials, n = 5 HIV-seronegative controls; 47% [9/19] male; median age, 32 years [IQR, 25-41]). Using this dataset, we determined analyte-specific positivity cut-points corresponding to an observed 10% false-positive-rate for the fold-change over background for each measured cytokine. Positivity cut-points related to a 10% false positive rate were unable to be established for IL-5 and fractalkine due to low response levels; therefore, the response rates for these two analytes were not calculated. To assess the sensitivity of this procedure using these positivity cut-offs, we measured the production of IFN-γ in PBMC samples from five HIV-infected individuals with known HIV-1 Gag-specific T-cell specificities; after applying our pre-determined IFN-γ positivity cut-off we observed a 100% true-positive rate in this group. Finally, we confirmed Luminex-determined concentrations of IFN-γ and IP-10 by ELISA in a subset of samples (R&D Systems, Minneapolis, MN).

### Statistical analyses

Statistical analyses were performed using SAS version 9.1.3 and JMP version 7.0 (SAS Institute, Cary, NC). Differences between Ad5 neutralizing antibody test groups (seronegative vs. seropositive) were tested using Wilcoxon rank tests, whereas comparisons of stimulations within the same individual were tested by Wilcoxon signed rank tests. Fisher's exact test was used to compare response rates, and Kruskal-Wallis was used to compare differences between stimulation conditions across analytes. All statistical comparisons were two-sided with an alpha level of 0.05. Only descriptive statistics were provided whenever a comparison group had less than five data points. Due to the exploratory nature of this study, all reported p-values were not subject to multiple comparison adjustments. However, to assess the impact of multiple comparisons on these reported results, we performed multiplicity adjustments by permutation or Hochberg methods to control the overall type I error (adjusted p-values not shown).

## Results

### Study Population and Multiplex Cytokine Assay Performance

The distributions of age and gender were well-matched between the two study groups (Table 1), but the Ad5-seronegative group had higher numbers of White participants compared to the Ad5-seropositive group (52.8% vs. 29.4%, p = 0.001 for all ethnicities). This general difference is consistent with the endemic seroprevalence of adenovirus serotype 5 inside versus outside the U.S. [22].

Peak T-cell responses were measured by IFN-γ ELISpot in PBMC isolated at 30 weeks after the first vaccination (4 weeks after the third vaccination) as part of the immunogenicity measurements of the phase I protocol. The study population had a median of 352 SFC/10^6^ PBMC, and the medians of the Ad5-seronegative and Ad5-seropositive groups were not significantly different (362 vs. 292 SFU/10^6^ PBMC, respectively; p = 0.42). To gain a broader understanding of vaccine-induced T-cell responses, we tested these samples for thirty secreted cytokines in response to Gag peptide stimulation using a multiplex cytokine assay that we developed. When we compared the IFN-γ response rates of the IFN-γ ELISpot and multiplex cytokine assays, we noted that 86% (48/56) of positive responders in the IFN-γ ELISpot assay had IFN-γ-secreting T cells detected by the multiplex cytokine assay (Table 2). The discordant results between the two assays may reflect a lower response frequency due to using different Gag peptide pools in each assay (Gag PTE peptides in the multiplex assay vs. homologous Merck peptides in the ELISpot assay), and also because the detection of individual IFN-γ-secreting cells by ELISpot may not absolutely correspond to extracellular protein secretion measured from multiple cells in a well.

### MRKAd5 HIV-1 gag vaccine produces a mixed Th1 and Th2 response profile of 11 cytokines in restimulated PBMC

**Figure 1:**
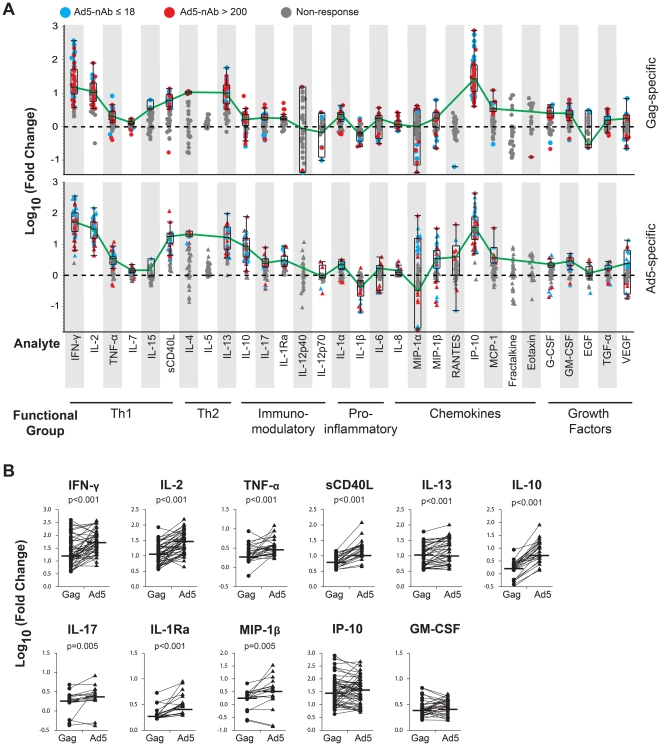
Peak vaccine response to Gag insert and Ad5 vector measured by *ex vivo* multiplex cytokine assay. Unfractionated PBMC from vaccinees collected at the peak response time point were assayed for 30 different cytokines and chemokines by multiplex bead array. Results are expressed as the log_10_ fold change in concentration compared to negative control wells. (**A**) Overall response profile of Ad5-seronegative (n = 28, blue dots) and Ad5-seropositive (n = 28, red dots) vaccinees to Gag (top) and Ad5 (bottom) for all analytes assayed. Non-responders are shown in gray. Box plots indicate interquartile ranges and medians for responders only, and medians are connected by the green line for profile comparison. (**B**) Comparison of the magnitude of up-regulation of the analyte focus set (selection criteria described in the text) in response to either Gag or Ad5. Groups were compared by Wilcoxon sign-rank. Horizontal lines denote median values.

We observed a wide range of T-cell response rates to Gag peptide pools at the peak time point across the thirty analytes tested. For example, all vaccine recipients tested had Gag-specific T cells that secreted IP-10, whereas T cells from only 2% (1/56) of participants with a Gag-specific response produced Regulated upon Activation, Normal T cell Expressed and Secreted protein (RANTES) or eotaxin (median response rate across analytes, 38%; IQR, 10.3-53.6%; see Table 2). After excluding responses below the positivity cut-off, the median magnitude of response over background (fold change) for the remaining vaccine recipients was highest for IP-10 (1.43 log_10_) and lowest for RANTES (−1.19 log_10_; Figure 1A, upper panel).

We focused our analysis on those analytes secreted by antigen-specific (Gag or Ad5) T cells that were detected in at least 50% of subjects with a significant difference of at least two-fold levels over or under background (Table 2, boldface). Based on these criteria, we identified a subset of eleven cytokines that were significantly expressed in this assay. Within this focus set, the greatest T-cell responses to Gag were the Th1-associated cytokines IP-10, IFN-γ, and IL-2, all which were detected in 86–100% of vaccine recipients tested. The Th2-associated cytokine IL-13 showed a median 12-fold increase in secretion over background in 71% of vaccine recipients, but the other Th2-associated cytokines, IL-4 and IL-5, were both below the level of detection. In addition, we found that following Gag stimulation, sCD40L, GM-CSF, TNF-α, IL-1Ra, IL-17, MIP-1β, and IL-10 were increased by 2-to-10-fold over background, with response rates of 34-70% (Table 2 and Figure 1A, upper panel). These results suggest that previously unrecognized immune factors associated with both Th1 and Th2 responses may contribute to the memory T-cell response induced by Ad5-HIV-1 vaccination.

### Higher IL-10 secretion distinguishes Ad5-specific from Gag-specific responses to vaccination

When evaluating PBMC from vaccine recipients in the multiplex cytokine assay, we found higher cellular response rates were produced by stimulation with Ad5 vector than with Gag peptides (Table 2). The overall response rate to Ad5 across all thirty analytes was 48% versus 40% to Gag peptides (Fisher's exact p<0.0001), and this difference in response rate was more evident when we limited the analysis to the previously-defined focus set of eleven analytes (82% Ad5-stimulated vs. 63% Gag-stimulated, Fisher's exact p<0.0001). In addition, compared to Gag-stimulation, Ad5 empty vector produced a significantly higher magnitude of response (p<0.0001) in every analyte of the focus set except for IP-10 (p = 0.53) and GM-CSF (p = 0.15) (Figure 1B).

Although cytokine profiling results showed that PBMC from vaccine recipients respond to both the insert and the vector with similar cytokine production profiles, Ad5 stimulation of PBMC produced significantly higher amounts of IL-10 than Gag stimulation. Therefore, we performed an overall test of the difference between Ad5- and Gag-specific responses between the eleven focus set analytes and observed a significant difference (Kruskal-Wallis, p<0.0001), with the difference in IL-10 secretion between Ad5- and Gag-stimulated PBMC representing the greatest difference (mean difference = log_10_(0.72), SD = 0.30) compared to the other 10 analytes (range of mean difference = log_10_(0.02)−log_10_(0.42)).

### Ad5- and Gag-specific IP-10 responses correlate with Ad5 serostatus

**Figure 2:**
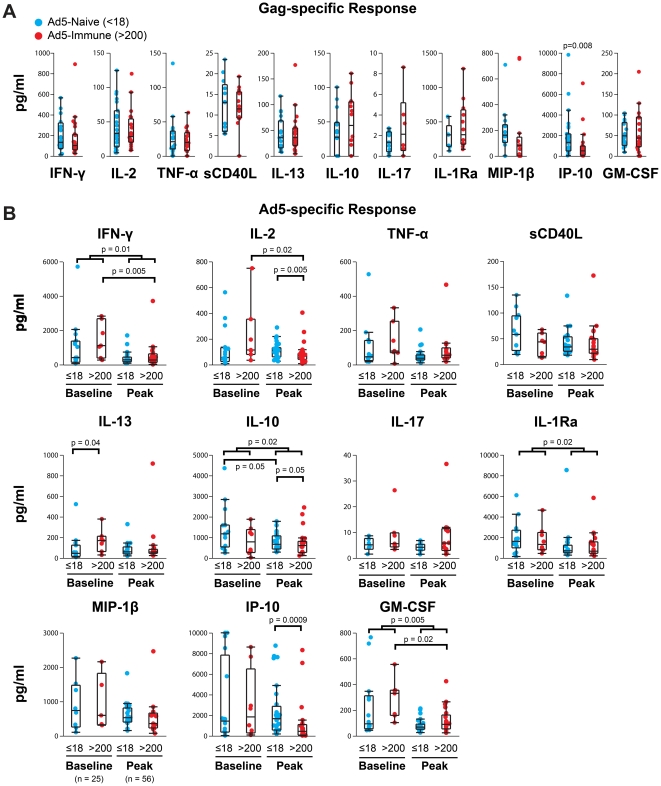
Comparison of Gag- and Ad5-specific cellular responses in Ad5-seronegative and Ad5-seropositive vaccinees. PBMC from vaccinees collected at a peak timepoint (28 weeks) were tested by multiplex cytokine assay. Background-subtracted concentrations of a focus set of eleven analytes are shown for Ad5-seronegative (blue) and Ad5-seropositive (red) test groups stimulated by either (A) a Gag PTE peptide pool or (B) Ad5 empty vector. Only positive responders, as reported in Table 2, were included in the analyses. Box and whisker plots indicate median and interquartile ranges, and groups were compared by Wilcoxon rank sums. Probability estimates are indicated when p≤0.05.

To determine the effects of pre-existing Ad5 neutralizing antibodies on Gag-specific responses, we compared the secreted amounts of Gag-induced cytokines and chemokines in the Ad5-seronegative and Ad5-seropositive test groups. Gag-stimulated PBMC from the Ad5-seronegative group secreted more than twice as much IP-10 as compared to the Ad5-seropositive group (median 1321 vs. 498 pg/ml, p = 0.008) (Figure 2A). In addition, Gag-stimulated PBMC from the Ad5-seronegative group, compared to the Ad5-seropositive group, secreted twice as much MIP-1β (160 pg/ml vs. 80 pg/ml, respectively), but this finding was not statistically significant (p = 0.08). When we measured the concentration of Ad5-specific cytokine secretions at the peak timepoint, we observed nearly four times as much IP-10 produced by PBMC from Ad5-seronegative as compared to Ad5-seropositive vaccinees (median concentration,1622 vs. 433 pg/ml; p = 0.0009; Figure 2B). Furthermore, in addition to the difference we observed for IP-10, Ad5 empty vector stimulation produced significantly higher concentrations of IL-2 and IL-10 in PBMC from the Ad5-seronegative group when compared to the Ad5-seropositive group (median 98 vs. 65 pg/ml; p = 0.005 and 545 vs. 342 pg/ml; p = 0.05, respectively). Likewise, following Ad5 stimulation, twice as much MIP-1β was seen in the Ad5-seronegative as compared to the Ad5-seropositive group (median 417 vs. 173 pg/ml, respectively), but this result was again not significant (p = 0.07). Taken together, these data suggest that the presence of pre-existing Ad5-neutralizing antibody titers may have a blunting effect that is qualitatively different for Ad5-specific immunity than for Gag-specific immunity.

### Ad5-specific cellular responses are detectable in the absence of Ad5 neutralizing antibodies

**Figure 3:**
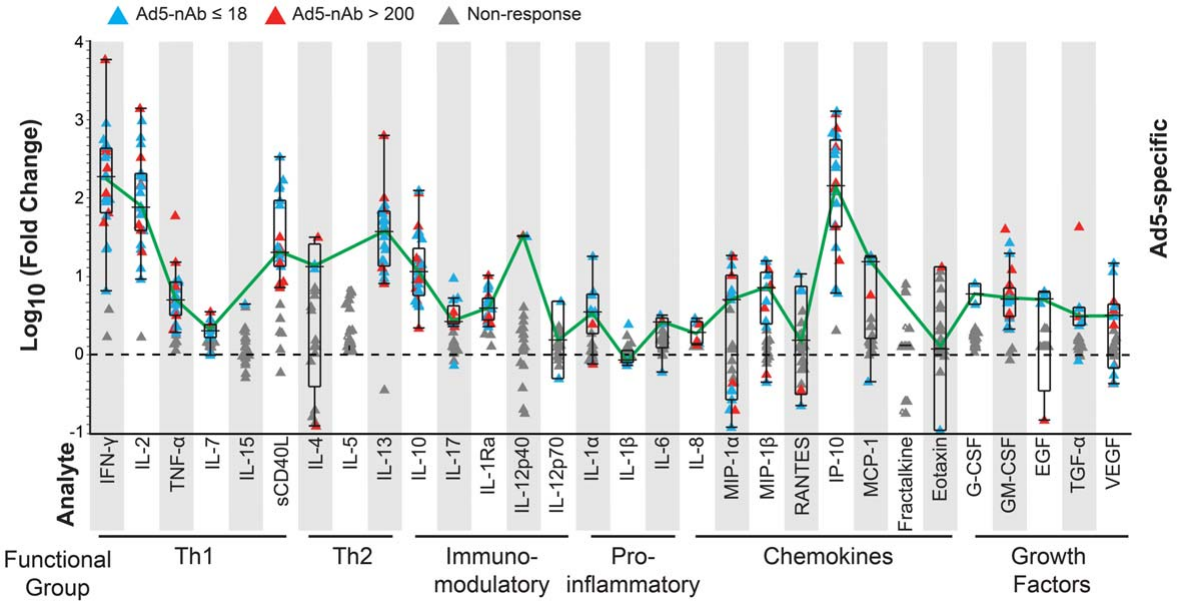
Frequency and magnitude of Ad5-stimulated cellular responses of vaccinees prior to vaccination. PBMC collected from trial participants prior to vaccination (baseline) were tested by multiplex cytokine assay. Magnitudes of response for 30 analytes tested are expressed as log_10_ fold change over background for Ad5-seronegative (n = 17, blue triangle) and Ad5-seropositive (n = 8, red triangle) volunteers. Non-responders are shown in gray. Box and whisker plots indicate median and interquartile ranges for responders only, while connecting line illustrates overall profile for comparison.

To determine if the Ad5-specific responses we observed in PBMC from Ad5-seronegative vaccine recipients were due primarily to the effects of vaccination with MRKAd5 HIV-1 *gag*, PBMC collected prior to vaccination were stimulated with Ad5 empty vector. We selected a subset of volunteers with sufficient available baseline samples (total n = 25: Ad5-seronegative, n = 17; Ad5-seropositive, n = 8) and included individuals in the peak time point testing (n = 11). Ad5-specific IFN-γ secretion was detected in PBMC from 92% (23/25) of donors collected at this pre-immunization time point (Figure 3). Of the two non-responders, one was Ad5-seronegative and the other was Ad5-seropositive. These unexpected results indicated that Ad5 serostatus was not correlated to the Ad5-specific cellular IFN-γ response based on our pre-defined positivity criteria for each assay (Fisher's exact test, p = 1.0). The only analyte that had a significant correlation between Ad5-specific cellular response rate and Ad5 serostatus was IL-17, which displayed an 87.5% response rate in the Ad5-seropositive test group (7/8) compared to a 35.3% response rate in the Ad5-seronegative group (6/17, Fishers exact test, p = 0.03; data not shown).

We next compared the Ad5-specific response profile at baseline to the profile from the peak immunogenicity timepoint and observed that a similar profile of cytokines was produced at both time points (Figures 1A, lower panel and Figure 3). Using the same criteria for determining a focused set of significantly up-regulated cytokines employed for the peak timepoint samples (i.e., greater than 50% response rate and greater than two-fold significant up- or down-regulation compared to controls), we found the same subset of 11 cytokines was selected at baseline as for the peak timepoints, and also observed that the relative levels of each cytokine in baseline samples were the same as in the peak time point samples. The defining characteristics of these similar cytokine profiles were that IFN-γ, IP-10, and IL-2 were produced in the greatest measured responses at baseline, followed in order by IL-13, sCD40L, IL-10, MIP-1β, GM-CSF, TNF-α, IL-17, and IL-1Ra (Figure 3). The cytokines absent from the peak timepoint responses were also not significantly up-regulated in baseline samples.

### Blunted Ad5-specific cellular responses correlate with boosted Ad5 neutralizing antibodies

After examining the naturally-occurring Ad5-specific immunity in vaccine recipients, we compared the overall (independent of Ad5 serostatus) concentrations of secreted cytokines from baseline and peak timepoint samples following Ad5 stimulation. We unexpectedly saw significantly higher concentrations of IFN-γ (p = 0.01), IL-10 (p = 0.02), IL-1Ra (p = 0.02), and GM-CSF (p = 0.005) were secreted from baseline samples when compared to peak time point samples (Figure 2B). We also observed a trend toward higher concentrations of TNF-α at baseline compared to the peak timepoint, but this difference was not statistically significant (p = 0.08). To determine if the cellular response to Ad5 empty vector following vaccination was more markedly reduced in vaccine recipients with pre-existing Ad5 neutralizing antibodies versus those without, we stratified our analysis into Ad5-seronegative and Ad5-seropositive test groups as described above. We found that PBMC from Ad5-seropositive vaccine recipients stimulated with Ad5 empty vector at baseline secreted four times more IFN-γ (median 1067 pg/ml vs. 267 pg/ml; p = 0.005) and GM-CSF (250 vs. 57 pg/ml; p = 0.02), and nearly twice much IL-2 (106 vs. 67 pg/ml; p = 0.02) compared to after vaccination (Figure 2B). In addition, we observed that PBMC from Ad5-seropositive vaccinees secreted two-to-four times less TNF-α (63 vs. 32 pg/ml; p = 0.08), IL-13 (158 vs. 55 pg/ml; p = 0.08), and IP-10 (1834 vs. 433 pg/ml; p = 0.07) after vaccination, but these latter results were not statistically significant (Figure 2B). Conversely, pre-vaccination PBMC from Ad5-seronegative vaccine recipients stimulated with Ad5 empty vector secreted about half as much IL-10 when compared to post-vaccination PBMC (545 vs. 1095 pg/ml, respectively; p = 0.05), but the levels of IFN-γ, IL-2, and GM-CSF did not differ in these comparisons.

**Figure 4:**
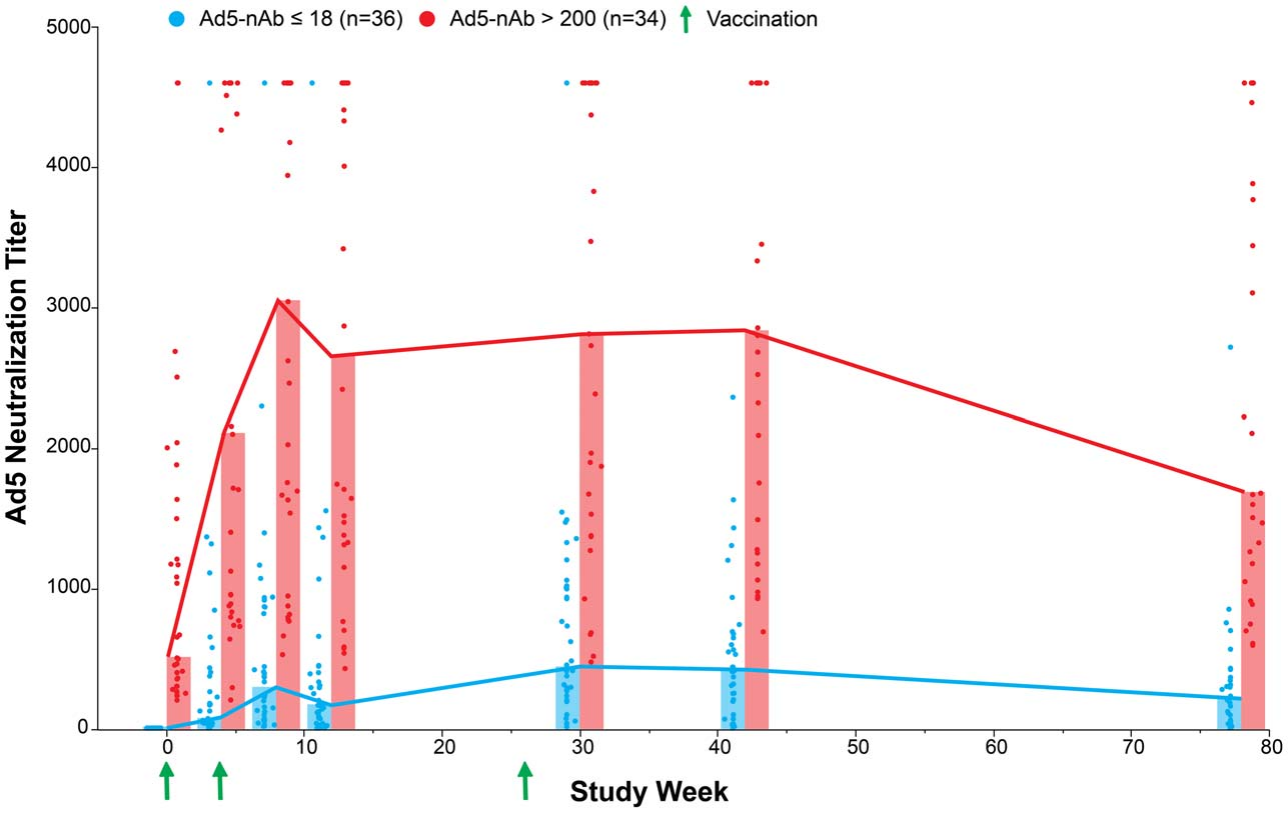
Ad5-specific neutralizing antibody titers before and after the MRKAd5 HIV-1 gag vaccine regimen. Serum samples collected at regular intervals during the vaccination schedule from Ad5-seronegative (n = 35, blue dots) and Ad5-seropositive (n = 34, red dots) vaccine recipients were assayed for Ad5 neutralizing antibody activity. Vaccination visits at 0, 4, and 26 weeks are indicated by green arrows. Bars indicate median neutralizing titers for each group at a given time point.

To test if the observed decrease in Ad5-specific cytokine production from PBMC following vaccination was related to increased humoral responses against the MRKAd5 HIV-1 gag vaccine, we measured the Ad5 neutralizing antibody titers in the test subjects from the baseline time point through 78 weeks of study follow-up. We observed that the median Ad5 neutralizing antibody titer was about six times higher in Ad5-seropositive group than the Ad5-seronegative group at the peak vaccine response timepoint (30 weeks, median 447 vs. 2821, p<0.0001) (Figure 4). Over the course of the 78-week follow-up period, the median difference in Ad5 neutralizing antibody titer ranged from six to 28 times higher in the Ad5-seropositive group and delineated differing plateaus based on pre-existing Ad5 neutralizing antibody levels. This contrasts with our results showing that Th1-associated cellular responses to the Ad5 vector were significantly lowered in the Ad5-seropositive group, but not the Ad5-seronegative group following vaccination.

Altogether, our results show that the cytokine profile of Gag-specific T-cell responses is remarkably similar to the vector-specific immunity that is present prior to vaccine administration, and the magnitude of insert-specific cytokine responses was associated with both humoral and cellular immune responses to the Ad5 vector.

## Discussion

The results of pre-clinical studies and the Step Study clearly indicate that more investigation is needed to clarify how pre-existing vector immunity can affect the outcome of vaccination against HIV-1 [1], [4]. We addressed this need in part by conducting a thorough evaluation of the insert- and vector-specific cytokine responses to an Ad5-HIV-1 Gag vaccine candidate similar to that used in the Step Study. Although recent studies have demonstrated that differences in Ad5-specific cellular immunity can affect vaccine-induced T-cell responses [2], [23], to our knowledge this is the first comprehensive analysis of HIV-1 vaccine-induced T-cell responses that has examined more than a few select cytokines. By surveying an extensive cytokine profile of antigen-specific T cells, we were able to observe that, in addition to IFN-γ, IL-2, and TNF-α, MRKAd5 HIV-1 gag vaccination induced Gag-specific T cells that secreted IP-10, IL-10, IL-13, sCD40L, GM-CSF, MIP-1β, IL-1Ra, and IL-17, most of which have not been previously related to HIV-1 vaccine-induced responses.

The remarkably similar cytokine profiles we observed in response to the Gag insert and the Ad5 vector suggests that the immune response to the vector will be predictive of the quality of T-cell response to the delivered immunogen. While the converse could not be formally ruled out in this study, other studies have shown that vector differences influence the type of immune response to an inserted transgene [4], [24], [25], [26]. In addition, our finding that the Ad5-specific cytokine profile is present prior to vaccination indicates that the type of response induced by a viral vector vaccine may be pre-determined by a vaccine recipient's memory repertoire defined by natural exposures and inherited HLA haplotype.

The lack of correlation between Ad5 serostatus and T-cell responses at baseline has been reported recently in other studies, in which increases in the frequency of IFN-γ and/or IL-2-secreting CD4^+^ and/or CD8^+^ T cells following Ad5/HIV vaccination in subjects with pre-existing Ad5 neutralizing antibody titers were also reported [23], [27], [28]. Of these studies, however, only one report included Ad5-specific responses past week eight (four weeks post-second vaccination), and that report's authors observed that the differences between Ad5 serostatus groups were transient and resolved after a third dose of vaccine [27]. In addition, all of these studies, including the present one, have only detected peripheral immune responses and not assessed immune response at mucosal surfaces or other sites of primary infection. It is likely that cross-reactivity to other adenovirus serotypes plays a role in the detection of T-cell responses to Ad5 empty vector in the absence of pre-existing Ad5 neutralizing antibodies, because there is significant conservation between members of adenovirus genera and a small number have now been shown to be cross-reactive in humans [28], [29], [30]. Although our data confirm the lack of correlation between Ad5 serostatus and Ad5-specific T-cell responses observed by others, in contrast to published reports we observed that levels of certain cytokines, such as IFN-γ, IL-10, GM-CSF and IL-1Ra, were reduced following vaccination. Furthermore, the levels of a different subset of cytokines (IL-2, IL-10 and IP-10) were lower in Ad5-seropositive compared to Ad5-seronegative vaccinees. This apparent disagreement with others' results may be due to the differences inherent in measuring the frequency of cytokine-producing cells by ICS and ELISpot versus measuring the actual amount of cytokine secreted by the multiplex cytokine assay used in our study. In addition, this assay as described is limited by the fact that it cannot distinguish between CD4^+^ and CD8^+^ T-cell responses and only a single post-stimulation timepoint was measured. Differences in assay characteristics highlight the need to examine multiple endpoints in the absence of a clear correlate of vaccine-induced immunity to HIV-infection or disease progression. In addition, it is important to note that differences we detected were not adjusted for any factors (e.g., age), and as expected, the significance levels were reduced after multiple comparison adjustments. However, although the results should be interpreted cautiously in consideration of the exploratory nature of this study, the overall conclusions of the study remain sound.

In the case of Ad5-specific immunity, the observed cytokine response profile was a complex mixture of Th1- and Th2- associated cytokine responses, and the higher IL-10 levels observed in response to the Ad5 empty vector compared to the Gag insert may be indicative of an immunosuppressive characteristic previously unrecognized in Ad5-based vaccine candidates or possibly related to the innate ability of adenovirus to activate macrophage and monocytes [31]. IL-10 is a pleiotropic immunomodulatory cytokine associated with immunosuppression that also, in combination with IL-4, IL-5, and IL-13, skews immune function *in vivo* towards a Th2 type of response [32]. The observation that IL-13 was produced in the presence of IFN-γ, TNF-α, and IP-10, raises questions about the efficacy of HIV-specific memory T-cell responses present when a mixture of Th1 and Th2 factors are engaged.

The additional contribution of IL-10 in this mixture of Th1 and Th2 responses remains to be clarified, but may be critical in light of recent studies that highlight a larger role of IL-10 in HIV-1 pathogenesis. In HIV-1 infected individuals, higher levels of IL-10 can blunt both the host T-cell response to HIV-1 and viral replication in macrophages and dendritic cells, as well as promote aberrant dendritic cell activity that may favor the virus [33], [34], [35]. Recent studies in mice have shown that blocking the IL-10 signaling pathway facilitates clearance of chronic viral infections [36], [37], and *in vitro* studies in PBMC from HIV-infected subjects have extended these findings to indicate that HIV-specific CD4^+^ and CD8^+^ T-cell responses are also augmented by antibodies that interrupt the IL-10/IL-10-receptor interaction [38]. Additional evidence that IL-10 expression levels may be important in providing protection against HIV-1 infection comes from the observation that genetic polymorphisms in IL-10 coding and promoter regions that are linked to higher IL-10 mRNA levels and associated with accelerated HIV-1 disease progression [39], [40]. In light of these interactions, an IL-10/IL-10-receptor blockade has been suggested as a potential adjunctive therapy for HIV-1 infection, with a proposed mechanism of viral clearance in the production and maintenance of functional antigen-specific memory T cells [41]. Since interruption of the effects of this immunosuppressive cytokine is beneficial for HIV-1 control, higher levels of IL-10 secretion in response to Ad5 stimulation may present a barrier to controlling of HIV-1 infection with Ad5-vaccine-induced T cells.

The fact that a significantly higher amount of IP-10 was produced in response to Gag peptides by PBMC from the Ad5-seronegative group when compared to the Ad5-seropositive group suggests that vaccine-induced antiviral cellular immunity may be affected by the presence of vector-specific neutralizing antibodies. IP-10 (interferon-γ-inducible protein, 10 kiloDaltons or CXCL10) is a chemoattractive cytokine which is rapidly produced in large amounts in response not only to IFN-γ, but also to other factors such as Type I interferon and lipopolysaccharide (reviewed in [42]). The fact that IP-10 production is most often seen in conjunction with IFN-γ presents the question of why IP-10 is the only cytokine that has significantly different regulation between vaccinees with or without pre-existing Ad5 immunity in our study. Two possible explanations for this discrepancy are that differing IP-10 levels are indicative of an antiviral amplification step that is affected by Ad5-specific immunity via an unknown mechanism, or alternatively, that vaccine-specific memory T cell populations may be differentially polarized to Th-helper cell subsets by the pre-existing vector-specific immunity. Support for the latter mechanism comes from data showing that the IP-10 receptor, CXCR3, is preferentially expressed on Th1-polarized cells in conjunction with other Th1-associated chemokine receptors, such as CCR5 and CCR2 [43]. Therefore, a decreased amount of IP-10 in Ad5-seropositive vaccinees may indicate that T cells are more likely to be polarized toward Th2, or other non-Th1 subtypes, such as the Th17 response. A trend towards a greater fraction of Ad5-seropositive than Ad5-seronegative volunteers producing IL-17 in response to Ad5 empty vector before vaccination may be indicative of such a Th17 polarization, but further studies will be necessary to confirm this connection.

Other previously unrecognized vaccine-induced cytokine responses produced in significant quantities by Gag- and Ad5-stimulated PBMC in our study were sCD40L (soluble CD40 ligand), IL-1Ra (interleukin-1 receptor antagonist), and GM-CSF (granulocyte-monocyte colony stimulating factor, or colony stimulating factor 2, [CSF2]). The net effect of this combination of cytokines in response to an Ad5-HIV-1 vaccine is unclear, but the individual contributions of each factor may provide clues. Previous work has shown that CD40L acts as a potent maturation agent for dendritic cell priming of antiviral CD8^+^ T cells [44]; it has recently been incorporated as an adjuvant for several viral vector vaccine candidates with some success [45], [46]. Whether or not such augmentation with CD40L can offer greater protection in an HIV/SIV challenge model has not been shown. Next, IL-1Ra is secreted in response to HIV-1 by macrophages *in vitro*, and increased plasma concentrations of IL-1Ra correlate with HIV-1 disease progression *in vivo* [47], [48]. However, a recent study of pig-tail macaques found IL-1Ra to be secreted at peak levels in the genital tract and plasma immediately following vaginal SHIV exposure, even if infection was not immediately productive [49]. This may indicate that innate stimulation of this immunosuppressive cytokine represents an advantage to HIV-1, thus limiting the production of IL-1Ra may be viewed as a beneficial outcome in future vaccine candidates. Finally, GM-CSF, which has long been associated with HIV-1 production in monocytes [50], [51], [52], was more recently introduced as an adjunctive therapy for leukopenia in HIV-1-infected individuals (reviewed in [53]) and was found to increase immune responses when incorporated as an adjuvant in certain next-generation HIV-1 vaccine candidates [54], [55]. Whether or not these cytokine responses can or should be enhanced further to boost the efficacy of an HIV-1 T-cell vaccine remains to be seen. However, this examination of the cytokine profile induced by an Ad5-HIV-1 vaccine may serve as a benchmark for comparing future vaccine candidates.

In contrast to the homologous prime-boost vaccine regimen used in this study, a recent challenge experiment in rhesus macaques showed that a heterologous prime-boost regimen with differing adenovirus strains produced more polyfunctional memory T cell populations and significantly lowered set-point viral loads [4]. Because of the cross-reactivity of cellular responses to different adenoviruses, this effect is likely due to a lack of induced humoral responses to the first vector administered (prime) that cross-react with the second vector (boost) [28], [29], [30], [56]. Although a general blunting of insert-specific responses due to pre-existing Ad5 immunity has been observed previously [57], [58], the results of our study extend this dampened response to cellular Ad5-specific responses following three doses of the MRKAd5 HIV-1 gag vaccine, while also demonstrating that the post-vaccination magnitudes of both Ad5 neutralizing antibodies and cellular responses are correlated with previous Ad5 exposure. Together, these results suggest that limiting vector exposures by administering fewer inoculations may be preferable, either with or without a heterologous prime, when designing a vaccine regimen.

Because IFN-γ, IL-2, and TNF-α production represent the canonical antiviral Th1 response, they are believed to be integral and necessary components of an effective anti-HIV-1 T-cell response. Although such responses may well be necessary, they are not sufficient to mitigate HIV-1 disease, as relatively high response rates and magnitudes of these cytokines were observed in the HIV-infected Step Study participants but they did not correlate with any observed reduction in viral load. By measuring the magnitude of an expanded panel of cytokines in future HIV-1 vaccine trials, we may find that protective immune responses are correlated with positive disease outcomes. Continuing to investigate immune parameters such as these will lead to a better understanding of the immune correlates of protection against HIV-1 disease progression and, hopefully, to an effective HIV-1 vaccine.
